# Cytomegalovirus reactivation and associated outcome of critically ill patients with severe sepsis

**DOI:** 10.1186/cc10069

**Published:** 2011-03-01

**Authors:** Alexandra Heininger, Helene Haeberle, Imma Fischer, Robert Beck, Reimer Riessen, Frank Rohde, Christoph Meisner, Gerhard Jahn, Alfred Koenigsrainer, Klaus Unertl, Klaus Hamprecht

**Affiliations:** 1 Klinik für Anaesthesiologie und Intensivmedizin, University Hospital of Tübingen, Hoppe-Seyler-Str.03, 72076 Tübingen, Germany; 2 Biostatistik-Tübingen, Burgunderweg 36, 72070 Tübingen, Germany; 3 Institut für Medizinische Biometrie, Westbahnhofstr. 55, 72070 Tübingen, Germany; 4 Institut für Medizinische Virologie und Epidemiologie der Viruskrankheiten, University Hospital of Tübingen, Elfriede-Aulhorn-Str. 6, 72076 Tübingen, Germany; 5 Klinik für Innere Medizin, University Hospital of Tübingen, Otfried-Müller-Straße 10, 72076 Tübingen, Germany; 6 Inzlingerstr.3, 79639 Grenzach-Wyhlen, Germany; 7 Universitätsklinik für Allgemeine, Viszeral- und Transplantationschirurgie, University Hospital of Tübingen, Hoppe-Seyler-Str.03, 72076 Tübingen, Germany

## Abstract

**Introduction:**

Sepsis has been identified as a risk factor for human cytomegalovirus (CMV) reactivation in critically ill patients. However, the contribution of CMV reactivation on morbidity and mortality is still controversial. Therefore, we analyzed the incidence and impact of CMV reactivation on outcome in patients with severe sepsis.

**Methods:**

In a prospective longitudinal double-blinded observational study, 97 adult nonimmunosuppressed CMV-seropositive patients with new onset of severe sepsis were included. Leukocytes, plasma and tracheal secretions were examined weekly for CMV-DNA by PCR. Tracheal secretions were additionally tested for HSV (Herpes Simplex Virus)-DNA. The influence of CMV-reactivation on the endpoints was analysed by Cox proportional-hazard regression analysis. Time-dependency was evaluated by landmark analysis.

**Results:**

Six out 97 died and five were discharged from the hospital within 72 hours and were excluded of the analysis. CMV reactivation occurred in 35 of the 86 (40.69%) analysed patients. HSV infection occurred in 23 of the 35 (65.7%) CMV reactivators. In 10 patients CMV-plasma-DNAemia appeared with a DNA-content below 600 copies/ml in four cases and a peak amount of 2,830 copies/ml on average. In patients with and without CMV reactivation mortality rates were similar (37.1% vs. 35.3%, *P *= 0.861), respectively. However, in the multivariate COX regression analyses CMV reactivation was independently associated with increased length of stay in the ICU (30.0, interquartile range 14 to 48 vs. 12.0, interquartile range 7 to 19 days; HR (hazard ratio) 3.365; 95% CI (confidence interval) 1.233 to 9.183, *P *= 0.018) and in the hospital (33.0, interquartile range 24 to 62 vs. 16.0, interquartile range 10 to 24 days, HR 3.3, 95% CI 1.78 to 6.25, *P *< 0.001) as well as prolonged mechanical ventilation (22.0, interquartile range 6 to 36 vs. 7.5, interquartile range 5 to 15.5 days; HR 2.6,CI 95% 1.39 to 4.94; *P *< 0.001) and impaired pulmonary gas exchange (six days, interquartile range 1 to 17, vs. three, interquartile range 1 to 7, days in reactivators vs. non-reactivators, *P *= 0.038). HSV reactivation proved not to be a risk factor for these adverse effects.

**Conclusions:**

These data indicate an independent correlation between CMV reactivation and increased morbidity in the well-defined group of nonimmunosuppressed patients with severe sepsis, but CMV reactivation had no impact on mortality in this group with low CMV-DNA plasma levels. Thus, the potential harms and benefits of antiviral treatment have to be weighed cautiously in patients with severe sepsis or septic shock.

## Introduction

Human cytomegalovirus (CMV) is widely recognized as the most serious viral pathogen in immunosuppressed patients, such as solid organ transplant recipients or those with malignant haematologic disorders or human immunodeficiency virus (HIV) infection [1-6]. Like other herpesviruses, CMV persists in the host after primary infection, usually remaining in a latent state for the rest of the host's life [2]. Disturbances in the balance between the host`s immune defenses and the non-active virus are thought to trigger CMV reactivation, which may result in CMV disease being associated with high morbidity and mortality in immunosuppressed patients [2,7].

Generally, critically ill patients in the intensive care unit (ICU) without exogenous immunosuppression are not thought to be endangered by CMV reactivation. However, in the last 10 years CMV reactivation rates close to those found after kidney transplantation have been observed in CMV-seropositive ICU patients, although the typical mechanisms of immunosuppression were absent [8-14]. In addition, there is a growing body of evidence that not only CMV but also herpes simplex virus (HSV) infections might have been considerably underestimated in critically ill patients [11,15]. The reactivation of both viruses is frequently observed in the respiratory tract, but there are few systematic studies investigating the reactivation of either CMV or HSV in respiratory tract specimens [11,16,17].

Single studies in different types of various ICU populations suggest that CMV reactivation might adversely affect the outcome of critically ill CMV-seropositive patients, independently of the occurrence of CMV disease, and in a similar fashion HSV infections might have negative effects on intensive care patients [9,13,17,18].

Bacterial sepsis has been identified as an independent risk factor for CMV reactivation in the heterogeneous population of critically ill patients [8,9]. Therefore, the question arises whether CMV infection contributes to increased morbidity and mortality to an extent warranting antiviral strategies in the risk group of patients with severe sepsis. To our knowledge, until now only one prospective study has addressed this issue in men [16], but statistical analysis could not be performed due to the limited collective of only 25 patients. Moreover, the role of coinfection with HSV in this context still remains to be elucidated. Therefore, we performed a prospective, blinded study monitoring nonimmunosuppressed, critically ill patients with severe sepsis for CMV reactivation in blood and also in respiratory secretions. Active HSV infection was evaluated as a potential cofactor of CMV infection. The aim of this investigation was to assess the impact of active CMV infection on survival, length of ICU and hospital stay as well as on duration of mechanical ventilation of non immunosuppressed patients with severe sepsis.

## Materials and methods

### Patients

This prospective observational study was performed in the surgical and the medical ICUs of the University Hospital Tübingen between February 2004 and September 2006. All adult patients of the two ICUs were daily screened for enrolment. The inclusion criteria were the presence of severe sepsis as defined by the consensus conference of the American College of Chest Physicians/Society of Critical Care Medicine (ACCP/SCCM) [19] and CMV seropositivity.

Exclusion criteria were the following: age younger than 18 years, pregnancy or breast feeding, duration of severe sepsis for longer than 72 hours, antiviral treatment with ganciclovir, valaciclovir, cidofovir, or foscarnet in the previous 7 days, and manifest immunosuppression because of HIV infection, congenital defects, leukopenia <2,000/μl, radiation or treatment with immunosuppressive substances within the last 6 months including prednisone, rituximab, alemtuzumab, tacrolimus, sirolimus, ciclosporin, mycophenolic acid, azathioprine, anti-lymphocytic or anti-IL6 antibodies.

### Study protocol

The investigation was approved by the local ethics committee of the Faculty of Medicine, which waived the need for informed consent. As soon as a patient fulfilled the inclusion criterion of severe sepsis and had no exclusion criterion present the first set of virological examinations including CMV serology was performed within the next three days.

Patients having a positive anti-CMV IgG titer were enrolled and further monitored for CMV reactivation once a week until discharge from the University Hospital or death.

Clinicians were not aware of the virological results, since they were assessed in a specific internal database applied for scientific purposes only. Ordering examinations to look for CMV disease as well as the initiation of antiviral treatment was left to the decision of the clinician, independently of the study.

The following data were evaluated at enrolment: age, gender, underlying disease requiring ICU treatment, the type of infection and the organ dysfunction constituting severe sepsis, presence of septic shock, the length of stay in the ICU, duration of mechanical ventilation and severity of illness and organ dysfunction as indicated by the Simplified Acute Physiology Score (SAPS) II [20] and the Sequential Organ Failure Assessment (SOFA) scores. Additionally, the records of each patient were reviewed for the presence of malignant disease, the number of surgical procedures, and the number of red blood cell units transfused during the current hospital stay before enrolment. After enrolment it was registered whether a CMV disease was diagnosed by the responsible clinicians. The study nurses collecting clinical data were blinded for virological findings with the exception of CMV serology, which was reported immediately.

The consequences of active CMV infection were longitudinally examined from enrolment until discharge or death by assessing in-hospital mortality, length of stay (LOS) in the ICU and in the hospital as well as time on mechanical ventilator.

### Virological assays

Samples were processed in the virological laboratory each Monday and Thursday independently from the day severe sepsis was diagnosed. The personnel performing the virological examinations had no contact with patients and no insight into clinical data. All data were fed into an internal database for scientific purposes to ensure mutual blinding. Longitudinal CMV monitoring included virus culture (human foreskin fibroblast monolayers) from tracheal secretions, qualitative nested PCR targeting the CMV IE1-Ex4 region [21] from leukocytes, plasma and tracheal secretions, and quantification of CMV-DNA (COBAS Amplicor CMV Monitor™ test, Roche Diagnostics, Mannheim, Germany) from qualitative PCR-positive plasma and tracheal secretion specimens. Experimental details are given elsewhere [22,23]. In the first virological examination CMV serology (anti-CMV IgG, anti-CMV IgM enzyme immunoassays from Medac, Wedel, Germany) was also assessed.

Moreover, respiratory secretions were examined by real time PCR using primers and hybridization probes derived from the DNA polymerase gene of HSV [24]. Vero cell monolayers were used to isolate HSV by cell culture. All CMV and HSV strains isolated from microculture were cryopreserved.

A status of viral latency was assigned if anti-CMV immunoglobulin G (IgG) was present but the virus could not be detected otherwise. Since earlier investigations had shown that healthy seropositive blood donors deliver negative CMV PCR results from leukocytes and plasma [25], CMV-DNA detection in plasma, leukocytes or respiratory secretions or positive virus isolation was defined as CMV reactivation.

### Statistics

Baseline patient characteristics were summarized using absolute frequencies and percentages with 95% confidence interval (CI 95%) for nominal data, and median (interquartile range (IQR)) for continuous data. The baseline characteristics were compared between the groups of patients with and without CMV reactivation using Fisher's Exact Test or Chi-Squared Test for nominal variables and Wilcoxon-Test for continuous variables. Patients who died or were discharged within the first 72 hours after study enrolment, were excluded from data analysis. The two primary endpoints were the rate of in-hospital mortality and length of stay in the ICU, defined as days from study enrolment to death or discharge from ICU. Secondary endpoints were duration of hospital treatment and length of mechanical ventilation defined accordingly. To evaluate the influence of CMV-reactivation on these endpoints we conducted uni- and multivariate Cox proportional-hazard regression analyses adjusting for confounding factors. The analyses regarding duration of hospital treatment and time on mechanical ventilation (secondary endpoints) were based on the data of the 55 surviving patients considering the following variables: SAPS II at inclusion (Score points), ICU stay before enrolment (days), septic shock at enrolment (yes/no) and HSV detection (duration of hospital treatment) and SAPS II, paO2/fiO2 ratio and presence of pneumonia causing sepsis at enrolment (yes/no) as well as duration of mechanical ventilation before inclusion (time on mechanical ventilation). Continuous variables were generally used as linear factors, all others were used as dichotomous factors in the regression models. Univariate hazard ratios were calculated with 95% CI (not shown in the tables) applying the Cox proportional-hazards model. The modelling included testing for co-linearity, interactions with the factor CMV reactivation, and proportional hazard assumption for the risk factors. In a first step the multivariate model considered all relevant risk factors, which were in a second step optimized keeping only CMV reactivation and those factors with a *P *< 0.05. Incidence figures were created using the Kaplan-Meier estimates. All *P-*values are two-sided. To additionally consider time-dependency a landmark analysis was performed at the time point 0, Day 7 and Day 14 based on the Cox-Regression. All statistical analyses were performed with SAS System version 9.1 for Windows (SAS Institute, Cary, NC, USA), and incidence figures were created with SPSS version 17.0 for Windows (SPSS Inc. Chicago, Illinois 60606, USA).

## Results

### Study population

A total of 129 patients were screened initially; 28 of them were excluded because of negative CMV IgG serology, 2 suffered from lymphoma, and 1 had to be excluded due to immunosuppressive chemotherapy. One patient was excluded because of missing data. Thus a total of 97 patients were enrolled for further CMV monitoring. Since six of them died and five were discharged from the hospital within 72 hours, the data of 86 patients were analysed; the majority of them (*n *= 64) were treated in the two surgical ICUs. Baseline demographic characteristics and clinical data of the 86 patients at enrolment are presented in Table 1.

**Table 1:** Demography and underlying conditions of included patients (*n *= 86)

	All patients	Active CMV infection		*P*-value
		Yes	No	
	*n *= 86 (100%)	*n *= 35 (40.69%)	*n *= 51 (59.31%)	
**Demographic data**				
Age (years)^a^	68.0 (59 to 76)	68.0 (52 to 73)	69.0 (59 to 76)	0.237
Male sex (n (%))	67 (77.9)	27 (77.1)	40 (78.4)	0.888
**Surgical interventions requiring intensive care (n)**				
Neurosurgery	4	2	2	
Abdominal surgery	33	14	19	
Cardiovascular surgery	18	7	11	
Other surgical procedures	9	6	3	
All surgical interventions	64	29	35	
**Medical diseases requiring intensive care (n)**				
Liver disease	1	1	0	
Coronary heart disease	2	0	2	
Infections in the internal ICU	16	4	12	
Other	3	1	2	
All medical diseases	22	6	16	0.137
**Infection causing severe sepsis (n (%))**^**b**^				
Pneumonia	22 (25.9%)	11 (31.4%)	11 (22.0%)	0.329
Peritonitis	34 (40.5%)	12 (35.3%)	22 (44.0%)	0.425
Urinary tract infection	7 (8.5%)	4 (12.5%)	3 (6.0%)	0.263*
Catheter-associated bacteremia	6 (7.3%)	3 (9.4%)	3 (6.0%)	0.435*
Other	38 (55.1%)	18 (66.7%)	20 (47.6%)	0.121
**Severity of illness at enrolment**				
Duration of ICU stay (days) ^c,d^	4.0 (2 to 9)	5.0 (2 to 11)	4.0 (2 to 9)	0.245
SAPS II Score^a^	43.0 (36 to 51)	43.0 (33 to 47)	44.0 (37 to 33)	0.150
SOFA Score^a^	8.0 (7 to 11)	8.0 (6 to 10)	9.0 (7 to 12)	0.060
Septic Shock present (n (%))	55 (65.5%)	19 (57.6%)	36 (70.6%)	0.221
Transfusion of packed red cells^a,d^	2.0 (0 to 6)	2.0 (0 to 5)	3.0 (0 to 8.5)	0.561
Duration of ventilation (days) ^c,d^	4.0 (2 to 9)	5.0 (2 to 11)	3.0 (2 to 9)	0.157
Surgical interventions ^a,d^	1.0 (1 to 2)	1.0 (1 to 2)	1.0 (1 to 2)	0.586
Horowitz index (paO_2_/fiO_2_) <200 (n (%))	56 (65.9)	23 (67.7)	33 (64.7)	0.779

^a^Median (interquartile range), ^b^All infections sum up to more than 100%, because more than one infection could be noted in one patient, ^c^Median (interquartile range), ^d^During the actual hospital stay until enrolment into the study * Fisher's exact test.

CMV, cytomegalovirus; SAPS, Simplified Acute Physiology Score; SOFA, Sequential Organ Failure Assessment score.

### Virological examination results

In the 86 study patients on average (median) four sets of samples for virological examination could be taken; 3.0 of them were collected during ICU stay, 3.0 on the ward.

**Figure 1:**
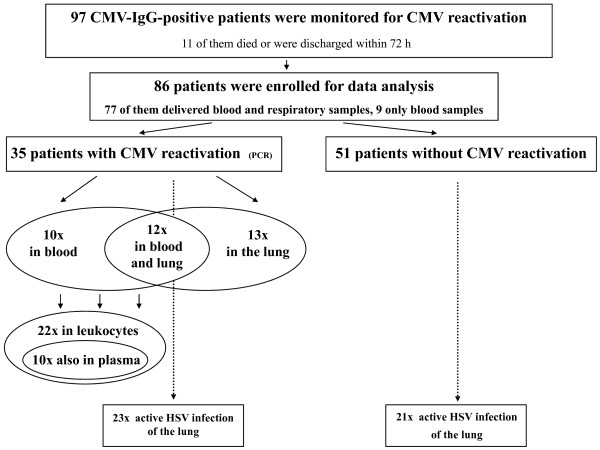
**Positive CMV and HSV PCR results in leukocytes, plasma, respiratory secretions of 97 patients**.

**Figure 2:**
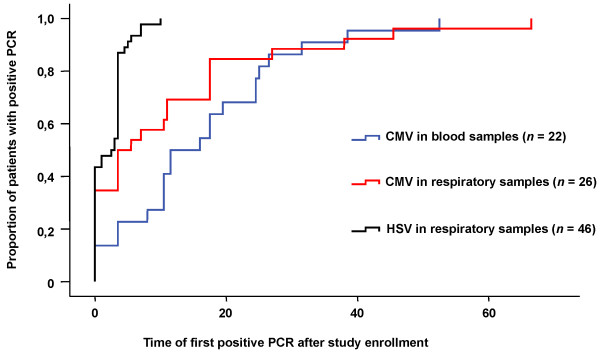
**CMV and HSV PCR in blood (CMV) and respiratory secretions against time after study enrolment**.

In 77 of the 86 patients both blood and tracheal secretions could be obtained for virological testing; 9 patients delivered only blood samples. Parameters of CMV reactivation were found in 35 of the 86 patients (40.7%, CI 95%: 30.2 to 51.8) with severe sepsis. The distribution of positive PCR results in the different compartments is presented in Figure 1, indicating that in 13 of the 35 cases CMV reactivation was detected exclusively in the lungs. On average (median) CMV reactivation occurred 21 days after enrolment into the study, becoming obvious earlier in tracheal secretions (median 14 days, range 0 to 77 days) than in blood (median 24.5 days, range 0 to 49 days), as shown in Figure 2. Interestingly, HSV-DNA appeared even more frequently and mostly earlier than CMV in respiratory secretions (Figures 1 and 2), yielding a positive PCR in 44 of the 86 study patients. In patients with CMV reactivation (*n *= 35) the rate of HSV detection added up to 65.7% (23 of 35) compared to 41.2% (21 of 51) in the group where CMV remained in the latent state (*P *= 0.025). Quantification of CMV-DNA was performed in the 10 patients who were tested positive in plasma by qualitative PCR. Four of them showed plasma DNA levels beyond the detection limit of the COBAS Amplicor^® ^PCR system (Roche) (600 copies/ml); in the other six patients the CMV-DNA content in plasma was low with a peak amount of 2,830 copies/ml on average (minimum 600, maximum 1,608 copies/ml). CMV-DNA in leukocytes was detected in 22 cases.

### Consequences of CMV reactivation

The in-hospital mortality of all enrolled patients was 36.1% (31 of 86) without any relevant difference between those who showed CMV reactivation (37.1%, that is, 13 of 35; CI 95% 21.5 to 55.1) and those who did not (35.3%, that is, 18 of 51, CI 95% 22.4 to 50.0; *P *= 0.861) (Table 2). No CMV disease was diagnosed by the responsible clinicians and thus no treatment was initiated. Even when adjusted for severity of illness, presence of septic shock, duration of ICU stay before study enrolment and HSV reactivation, in-hospital mortality of patients with CMV reactivation was not increased (HR: 0.369, 95% CI: 0.136 to 1.005, *P *= 0.051 Table 3). To light up time-dependency of the CMV effect on in-hospital mortality, we applied Cox regression modelling at days 0, 7 and 14 (landmark analysis) considering the same factors thereby including HSV detection according to its occurrence at the three time points. At each time point, interaction between the tested factors was proven to be not statistically significant. Results of the optimized models are shown in Table 3. These data confirm that only SAPS II at inclusion influenced the in-hospital mortality.

**Table 2:** Outcomes of included patients with and without CMV reactivation (*n *= 86)

		All patients	Active CMV infection		*P*-value
			Yes	No	
Parameter	n	*n *= 86	*n *= 35	*n *= 51	
Mortality (n (%))*	86	31 (36.1)	13 (37.1)	18 (35.3)	0.861
Length of stay in the ICU (days) ^a, b ^*	86	16.5 (7 to 29)	30.0 (14 to 48)	12.0 (7 to 19)	<0.001
Length of hospital stay (days) ^a, b ^**	86	22.5 (13 to 38)	33.0 (24 to 62)	16.0 (10 to 24)	<0.001
Duration of mechanical ventilation (days) ^a, b ^**	82	12.0 (6 to 23)	22.0 (6 to 36)	7.5 (5 to 15.5)	0.003

^a ^Median (interquartile range); ^b ^Defined as days after study enrolment

* Primary endpoints; ** Secondary endpoints. CMV, cytomegalovirus.

**Table 3:** Cox regression analyses of factors associated with in-hospital mortality of the 86 included patients

	Univariable analysis		Multivariable analysis		
Factor	HR	*P-v*alue	HR	95% CI	*P-v*alue
CMV reactivation	0.410	0.029	0.369	0.136 to 1.005	0.051
SAPS II at inclusion	1.062	<0.001	1.047	1.012 to 1.082	0.008
Septic Shock present	2.193	0.081	1.470	0.555 to 3.896	0.438
ICU stay before enrolment	1.010	0.298	1.022	0.999 to 1.046	0.058
HSV detection in respiratory secretions	1.539	0.268	1.546	0.687 to 3.480	0.292
**Optimized model**					
CMV reactivation			0.496	0.215 to 1.145	0.101
SAPS II at inclusion			1.056	1.024 to 1.089	<0.001
**Landmark analysis for Day 0, Day 7 and Day 14**					
**Day 0 **(86 patients, 12 of them with CMV reactivation)					
CMV reactivation	0.983	0.974	1.005	0.347 to 2.911	0.993
SAPS II at inclusion	1.062	<0.001	1.062	1.030 to 1.096	<0.001
**Day 7 **(75 patients, 18 of them with CMV reactivation)					
CMV reactivation	0.559	0.259	0.486	0.173 to 1.370	0.172
SAPS II at inclusion	1.063	0.001	1.064	1.027 to 1.102	<0.001
**Day 14 **(62 patients, 20 of them with CMV reactivation)					
CMV reactivation	0.707	0.492	0.561	0.204 to 1.544	0.263
SAPS II at inclusion	1.065	0.003	1.067	1.026 to 1.111	0.001

CMV, cytomegalovirus; HR, hazard ratio; HSV, herpes simplex virus; SAPS, Sequential Organ Failure Assessment score.

Focussing on increased morbidity an association with CMV reactivation was observed. The LOS in the ICU (30.0, interquartile range 14 to 48 vs. 12, interquartile range 7 to 19 days; *P *< 0.001) as well as the duration of hospital treatment (33.0, interquartile range 24 to 62 vs. 16.0 days, interquartile range 10 to 24; *P *< 0.001) and the time on mechanical ventilation (22.0, interquartile range 6 to 36 vs. 7.5 days, interquartile range 5 to 15.5; *P *= 0.003) were significantly longer in patients with CMV reactivation than in those without (Table 2).

The impact of CMV reactivation on the LOS in the ICU was further elucidated by Cox regression again considering the factors mentioned above (Table 4). This Cox model showed that CMV reactivation was clearly associated with a longer ICU stay (HR 3.365, CI 95% 1.233 to 9.183; *P *= 0.018 and HR 2.441, CI 95% 1.011 to 5.897, *P *= 0.047, respectively, according to the optimized model). Moreover, as for in-hospital mortality a landmark analysis was performed based on the same risk factors looking forward on the length of ICU stay following days 0, 7 and 14 after enrolment, respectively (Table 4). The SAPS II at inclusion became statistically significant at all three time points and proved to be the most important risk factor for prolonged ICU treatment. In this landmark analysis only CMV reactivation at Day 7 was identified as a second risk factor with an independent impact on the length of ICU stay following Day 7 after study inclusion (HR 2.853, CI 95% 1.003 to 8.117, *P *= 0.049).

**Table 4:** Cox regression analyses of factors associated with LOS in the ICU of the 86 included patients

	Univariable analysis		Multivariable analysis		
Factor	HR	*P-v*alue	HR	95% CI	*P-v*alue
CMV reactivation	3.101	0.009	3.365	1.233 to 9.183	0.018
SAPS II at inclusion	1.065	<0.001	1.045	1.008 to 1.083	0.016
Septic Shock present	2.282	0.081	1.486	0.535 to 4.127	0.447
ICU stay before enrolment	1.006	0.577	1.020	0.996 to 1.044	0.104
HSV detection in respiratory secretions	1.302	0.501	1.338	0.595 to 3.013	0.481
**Optimized model**					
CMV reactivation			2.441	1.011 to 5.897	0.047
SAPS II at inclusion			1.055	1.020 to 1.091	0.002
**Landmark analysis for Day 0, Day 7 and Day 14**					
**Day 0 **(86 patients, 12 of them with CMV reactivation)					
CMV reactivation	1.017	0.975	1.061	0.367 to 3.068	0.913
SAPS II at inclusion	1.065	<0.001	1.065	1.030 to 1.101	<0.001
**Day 7 **(64 patients, 16 of them with CMV reactivation)					
CMV reactivation	2.274	0.112	2.853	1.003 to 8.117	0.049
SAPS II at inclusion	1.071	0.001	1.074	1.033 to 1.116	<0.001
**Day 14 **(47 patients, 17 of them with CMV reactivation)					
CMV reactivation	2.109	0.164	2.538	0.861 to 7.479	0.091
SAPS II at inclusion	1.065	0.010	1.069	1.021 to 1.120	0.004

CI, confidence interval; CMV, cytomegalovirus; HR, hazard ratio; HSV, herpes simplex virus; LOS, length of stay; SAPS, Sequential Organ Failure Assessment score.

**Figure 3:**
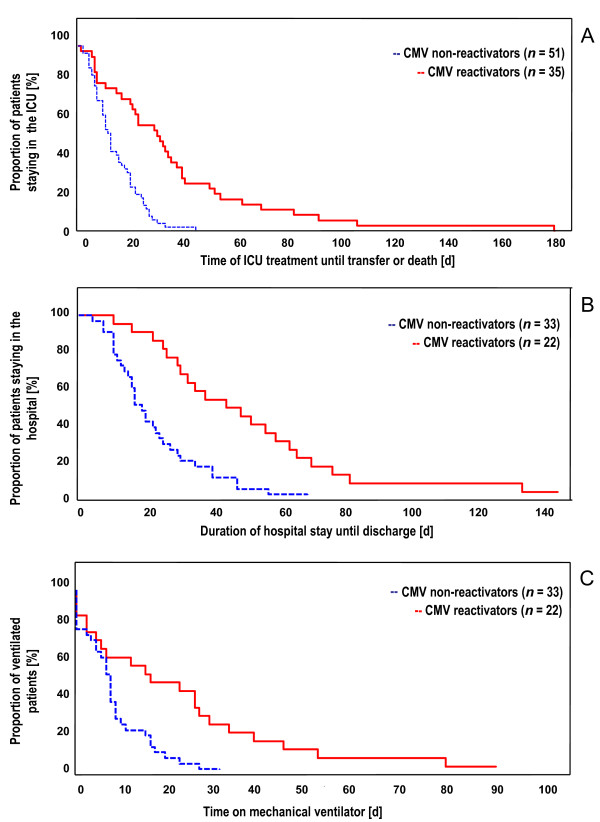
**A: Patients leaving ICU due to transfer or death against days after study enrolment**. **B**: Surviving patients discharged from hospital against days after study enrolment. **C**: Patients on mechanical ventilation against days after study enrolment.

Moreover the surviving patients with CMV reactivation were at significantly higher risk for prolonged in-hospital treatment as shown in Figure 3 (HR 3.3; CI 95% 1.78 to 6.25; *P *< 0.001). The adjusted proportional hazard ratio for prolonged mechanical ventilation was 2.6 times higher in CMV reactivating patients than in those who remained in a latent state (CI 95% 1.39 to 4.94, *P *< 0.001; optimized model; Figure 3). The increased time of mechanical ventilation went along with a significantly compromised pulmonary function in patients with CMV reactivation. The Horowitz index (paO_2_/fiO_2 _ratio) remained below 200 for six days (interquartile range 1 to 17) in CMV reactivators compared with three days in non-reactivators (interquartile range 1 to 7, *P *= 0.038).

## Discussion

This prospective, observational study demonstrated CMV reactivation in 40.69% (35 of 86) of patients with severe sepsis, despite the absence of other factors causing immunosuppression. This incidence of CMV reactivation is amazingly consistent with the results of two previous small German studies [12,16] and a more recent retrospective investigation [26]. These authors calculated a CMV reactivation rate of 45% in patients with systemic inflammatory response syndrome or sepsis [12], of 32% in patients with septic shock [16] and of 35% in cryopreserved plasma samples of long-term ICU patients [26]. Thus, as proposed in the review by Osawa and Singh [27], our prospectively assessed data clearly identify septic patients as a defined subgroup in the ICU population being at high risk for CMV reactivation.

A study examining 120 CMV-seropositive patients in six US ICUs also revealed CMV reactivation in approximately one-third of the study group [13]. There is, however, an important difference; whereas Limaye and co-workers [13] defined CMV reactivation exclusively on the basis of findings in plasma, our study additionally considered findings in leukocytes and respiratory secretions. Referred to positive PCR results in plasma only, the CMV reactivation rate in our study group was clearly lower (11.6%) than in Limaye's population.

A recent French investigation of 242 patients in a medical ICU [9], which evaluated both blood and respiratory samples indicated active CMV infection in 16% of the patients, which is less than half of the CMV reactivation rate observed in our patient population. This discrepancy can be attributed to differing CMV detection methods, since the French results rely on virus isolation from respiratory secretions. This technique has been shown to be less sensitive than PCR-based methods [18,27] as used in our study. Another explanation might be a lower CMV risk of medical compared with surgical ICU patients in general [14,27]. This difference was also reflected in our own study group with a CMV reactivation rate of 27.3% versus 45.3% (*P *= 0.137) in medical and surgical patients, respectively.

In general any comparison of incidence rates for CMV reactivation in critically ill patients is still compromised by differences in the examined materials, by the use of various virological methods [14,26] and most importantly, by differences between patient populations, which are not reflected by usual scores as for example SAPS II or SOFA.

Our finding that HSV reactivation appeared in nearly half of the patients and was thus clearly more frequent than CMV infection (Figure 2) agrees closely with the data of Cook *et al*., who reported positive HSV and CMV cultures in 23% and 15% of critically ill surgical patients, respectively [11]. The frequent coincidence of both herpes virus infections appeared quite similarly in a small study group of 25 septic patients, where 6 of the 8 CMV-reactivating patients showed active HSV infection as well [16].

Mortality rates did not differ between patients with and without CMV reactivation in our study group. Slight differences between the two patient groups at baseline regarding SAPS II, presence of septic shock and ICU stay before enrolment suggest that selection bias might have contributed to this finding. This limitation due to the observational design of our study has to be taken into account. To address this problem a Cox regression adjusting for other potential risk factors was conducted. But even this adjusted analysis showed no impact of CMV reactivation on mortality (Table 3). This finding may surprise at the first glance, because recent results obtained in patients of French and US ICUs [9,13] as well as our own earlier findings in surgical ICU patients [8] suggested a higher mortality rate in CMV reactivators. The main reason for this discrepancy might be the difference between the homogenous group of patients with severe sepsis or septic shock presented here and the more heterogeneous cohorts of ICU patients enrolled in the other studies. This assumption is strongly corroborated, when our own previous results [8] are compared with the actual findings. Although identical methods were applied in the same setting, a remarkable effect of CMV reactivation on mortality appeared in the mixed group of seropositive surgical ICU patients of our former study, but not in the current one exclusively focussing on patients with severe sepsis. In this patient population, severity and treatment of sepsis might be the most crucial prognostic factors overriding potential effects of CMV reactivation. Beyond this, the extent of the viral load might strongly determine the effects on patient outcome, as suggested by Limaye's data [13] and also by the observations of Linssen and coworkers [28] in ICU patients with HSV detection in respiratory secretions. This assumption is strongly corroborated by the comparison of our findings with Limaye's results. Indeed, quantitative PCR examinations delivered >1,000 copies per ml plasma in 8% of our patients but in 20% of Limaye's patients. The higher incidence of plasma-DNAemia as well as the higher level of plasma DNAemia observed by Limaye *et al*. indicates that their patients developed a more serious pattern of CMV reactivation than ours. Unequal treatment modalities, such as transfusion of leukocyte-depleted versus non-depleted blood products or different catecholamine use [29] might have contributed to the different severity of CMV reactivation and, thereby, to different effects on in-hospital mortality.

Finally, it has to be mentioned, that a small effect of CMV reactivation on mortality could have been overseen in our study due to the restricted number of examined patients. This is a major limitation of our study, delivering a statistical power of less than 20% to detect a 10% mortality difference between CMV reactivators and non-reactivators.

CMV reactivation in our patients with severe sepsis was accompanied by an increased LOS in the ICU (30.0, interquartile range 14 to 48 vs. 12, interquartile range 7 to 19 days; *P *< 0.001), an extended time of in-hospital treatment (33.0, interquartile range 24 to 62, vs. 16.0, interquartile range 10 to 24 days; *P *< 0.001), and longer time on the ventilator (22.0, interquartile range 6 to 36, vs. 7.5 interquartile range 5 to 15.5 days; *P *= 0.003) (Table 2). However, these data alone cannot give a clue on the causality of CMV reactivation. The first reason is that other risk factors rather than CMV reactivation might have led to these enhanced treatment requirements. To address this point, we adjusted for the most probably relevant factors using Cox regression (Table 4) but the number of included factors had to be limited to four (beyond CMV reactivation) in order to avoid overfitting of the model [30], which is a limitation due to sample size in our study. Nevertheless, the adjusted analysis confirmed the significant impact of CMV reactivation on the LOS in the ICU (primary endpoint) as well as on the duration of in-hospital treatment and time on mechanical ventilator (secondary endpoints). The other result of the Cox regression, delineating HSV in contrast to CMV not as a risk factor for prolonged ICU stay, fits well with an earlier finding of Tuxen *et al*. [31], who found no positive effect of acyclovir prophylaxis on the LOS in ICU.

A second problem questioning the causative role of CMV reactivation for prolonged intensive care treatment is the potential confounding of LOS in the ICU with opportunity to detect CMV. Unfortunately, a major limitation of our study was that, due to logistical reasons, CMV monitoring could not be continued after hospital discharge of the patients. Therefore, in an attempt to address this important issue, a landmark analysis was also performed. This evaluation corroborated the association of CMV reactivation with prolonged ICU stay when we looked forward from Day 7 on the subsequent LOS. Since this statistical approach allows control for time-dependency of an effect it strengthened the assumption, that CMV reactivation might be a true causative factor contributing to extended treatment needs in patients with severe sepsis. This suggestion is corroborated by the findings in various mixed ICU populations [9,13,17,18] and in one small population of 25 septic patients [16].

In severely immunosuppressed patients like in stem cell transplantation, CMV pneumonia may lead to fatal outcome. CMV disease was reported in single cases of acutely ill, but otherwise immunocompetent, patients [32], but such cases are rare and were not observed in this study. Thus, other effects of CMV must be responsible for our findings. Although the incidence of acute respiratory distress syndromes was not specifically addressed in our study; impaired pulmonary function might be a possible explanation. In patients with CMV reactivation, impairment of the pulmonary gas exchange (paO2/fiO2 <200) persisted significantly longer than in non-reactivating patients (6.0, interquartile range 1 to 17 vs. 3.0, interquartile range 1 to 7 days, *P *= 0.038. This result corresponds well with the findings of Cook *et al*. [33] obtained in a mouse model of CMV reactivation due to sepsis.

Nevertheless, one must keep in mind that the above mentioned limitations of our study design do not allow us to unequivocally establish the causative role of CMV for extended treatment requirements. We cannot exclude the possibility that CMV reactivation could be a marker, rather than a cause, of serious illness.

As proposed by Osawa and Singh [27], a prospective randomised multicenter trial of prophylactic antiviral treatment might be the most goal-oriented method to establish the causative role of CMV in adverse outcomes. The fact that Limaye *et al*. observed a quantitative association between CMV reactivation in terms of plasma CMV-DNA levels and a combined endpoint (death or ICU stay beyond day 30), whereas survival was unaffected in our patients, who had much lower plasma DNA levels, corroborates the importance of quantitative examinations. A quantitative approach might offer the chance to optimize the balance of potential harms and benefits for participants of a randomized treatment trial.

## Conclusions

In summary, our data indicate an independent correlation between CMV reactivation and increased morbidity in the well-defined group of nonimmunosuppressed patients with severe sepsis, but CMV reactivation had no impact on mortality in this group with low CMV-DNA plasma levels. Thus, the potential harms and benefits of antiviral treatment have to be weighed very cautiously in patients with severe sepsis or septic shock.

## Key messages

• Cytomegalovirus reactivation occurs in 40% of non-immunosuppressed CMV-seropositive critically ill patients with severe sepsis.

• Cytomegalovirus reactivation had no relevant impact on mortality but was associated with increased length of stay in the ICU and in the hospital.

• Cytomegalovirus reactivation was accompanied by Herpes simplex infection in 65.7% of cases.

• Herpes simplex occurs earlier than CMV reactivation during severe sepsis.

## Abbreviations

ACCP: American College of Chest Physicians; CI: confidence interval; CMV: cytomegalovirus; HR: hazard ratio; HSV: herpes simplex virus; LOS: length of stay; SAPS: Simplified Acute Physiology Score; SCCM: Society of Critical Care Medicine; SOFA: Simplified Organ Failure Assessment.

## Competing interests

This study was sponsored in part by Roche Pharma AG, Grenzach-Wyhlen, Germany. One of the co-authors (FR) was formerly employed by Roche Pharma AG, whose product is active against CMV infection, which was studied in the present work. There are no other competing interests.

## Authors' contributions

AH designed and carried out the clinical study including data analysis, and was the responsible first author, with an unrestricted grant as mentioned above. HH recruited samples and is the corresponding author. IF was responsible for statistical analysis. RB performed the virological analysis (HSV PCR) and data analysis. RR recruited patients. FR was responsible for discussion and the literature search. CM was responsible for statistical analysis. GJ interpreted the data. AK and KU recruited patients and contributed to the discussion. KH co-designed the virological study and was responsible for virological monitoring, including the data analysis.
